# Process-Structure-Property Relationships of AISI H13 Tool Steel Processed with Selective Laser Melting

**DOI:** 10.3390/ma12142284

**Published:** 2019-07-16

**Authors:** Morteza Narvan, Kassim S. Al-Rubaie, Mohamed Elbestawi

**Affiliations:** Department of Mechanical Engineering, McMaster University, 1280 Main Street, West Hamilton, ON L8S 4L7, Canada

**Keywords:** H13 tool steel, selective laser melting, volumetric energy density, relative density, surface roughness, microstructure

## Abstract

Due to a good combination of high hardness, wear resistance, toughness, resistance to high operating temperatures, and fairly low material cost, AISI H13 tool steel is commonly used in the manufacture of injection molds. Additive manufacturing (AM) such as selective laser melting (SLM), due to the layer-wise nature of the process, offers substantial geometric design freedom in comparison with conventional subtractive manufacturing methods, thereby enabling a construction of complex near-net shape parts with internal cavities like conformal cooling channels. The quality of SLM-manufactured parts mainly depends on the part geometry, build orientation and scanning strategy, and processing parameters. In this study, samples of H13 tool steel with a size of 10 × 10 × 15 mm^3^ were SLM-manufactured using a laser power of 100, 200, and 300 W; scanning speed of 200, 400, 600, 800, 1000, and 1200 mm/s; and hatch spacing of 80 and 120 µm. A constant layer thickness of 40 µm, 67° scanning rotation between subsequent layers, and a stripe scanning strategy were maintained during the process. The samples were built considering a preheating of 200 °C. The relative density, surface roughness, crack formation, microstructure, and hardness were evaluated. The relative density is shown to increase with increasing the volumetric energy density up to a value of about 60 J/mm^3^ and then no significant increase can be pointed out; the maximum relative density of 99.7% was obtained. A preheating of 200 °C generally aids to increase the relative density and eliminate the crack formation. The microstructure of built samples shows fine equiaxed cellular-dendritic structure with martensite and some retained austenite. The microhardness of the as-built samples was found to vary from 650 to 689 HV 0.2, which is comparable to a conventionally produced H13 tool steel.

## 1. Introduction

Owing to the layer-wise nature of the process, additive manufacturing (AM) technology allows with a high degree of accuracy for the manufacture of complex-shape geometries, quite difficult or impossible to obtain using conventional material-removal processes, hence opening significant opportunities up for the design of novel geometries and complex internal structures. The manufacture of a given part using AM technique is based on the slicing its 3D CAD model into multiple layers, creating a tool path for each layer, uploading the data in the AM machine, and building the part up layer by layer, following the sliced model [1,2]. To build a layer of predefined geometry, the powder is melted by a focused heat source provided by an electron beam, laser, plasma or electric welding arc, etc. The fabrication of a component by AM technology does therefore aid to eliminate the need for molds and dies or any additional fixtures, coolants and cutting tools, with minimal finishing operations, resulting in a significant reduction in lead-time, material wastes, energy, and costs. Due to its vast advantages, AM has become a crucial alternative manufacturing technique for small quantities of components having complex geometries [1]. In addition to polymers, ceramics, and composites, a variety of metallic materials can be fabricated by AM. Of several AM processes, selective laser melting (SLM) has gained an essential role in the field of metallic materials. SLM implies that a laser beam selectively melts and fuses accumulating layers of powder. 

The SLM technique has recently attracted the attention of the tool and die manufacturers, owing to the possibility of producing tool inserts with sophisticated features like conformal cooling channels [3,4,5]. The idea behind the conformal cooling system is to conform the 3D geometry of the cooling channels to the contours of the part, aiming at maximizing heat dissipation, uniform cooling, ultimately, higher productivity. However, the response of the conventional alloys to the acute conditions experienced in the process could restrict full implementation of conformal cooling systems. In comparison to the traditional manufacturing routes, materials fabricated by SLM display distinctive microstructures, being direct result of the interaction of a focused high energy laser beam with the material that leads to high heating and cooling rates, rapid solidification, and large thermal gradients within the melt pools [6]. By far, despite the high interest in this technology, only few alloys have been processed reliably by the SLM [7] and, in particular, very limited studies have been published on the high strength steels for tooling. Some studies have targeted maraging steels, investigating the material processing and the effects of post-process heat treatments on the microstructure and mechanical properties [8,9,10]. In other studies, processing parameters and microstructural features of SLM-manufactured M2 high speed tool steel, some grades of cold-work tool steels, and H11 hot-work tool steel have been investigated [11,12,13,14,15,16].

Due to a good combination of high hardness, wear resistance, toughness, resistance to high operating temperatures and thermal fatigue, and fairly low material cost, AISI H13 hot-work tool steel is commonly used in the manufacture of injection molds. H13 tool steel finds its core applications in processes such as plastic injection molding, die casting, forging, and extrusion [17]. H13 tool steel exhibits a complex processing behavior owing to its high hardenability resulting from the high carbon level and alloying elements. The change in specific volume during phase transformation in the solid state can bring about additional stresses, consequently, promoting crack propagation and distortion. Table 1 shows an overview on the SLM machines, processing parameters, and some remarks pointed out by the researchers.

Although SLM has benefited the industry by providing the designers with a significant freedom in design, its full implementation is restricted by common defects generated during the process. Porosity, cracking, surface roughness, loss of alloying elements, and residual stresses are commonly known defects in SLM [18]. Understanding the behavior of the material in response to the processing parameters and subsequently finding a safe processing window to avoid these defects is paramount. This work aims to study the behavior of SLM-manufactured H13 hot-work tool steel under a wide variety of processing parameters. Moreover, preheating is investigated as an effective way to diminish the developed defects during the process.

## 2. Experimental Procedures

### 2.1. Powder Material 

**Figure 1:**
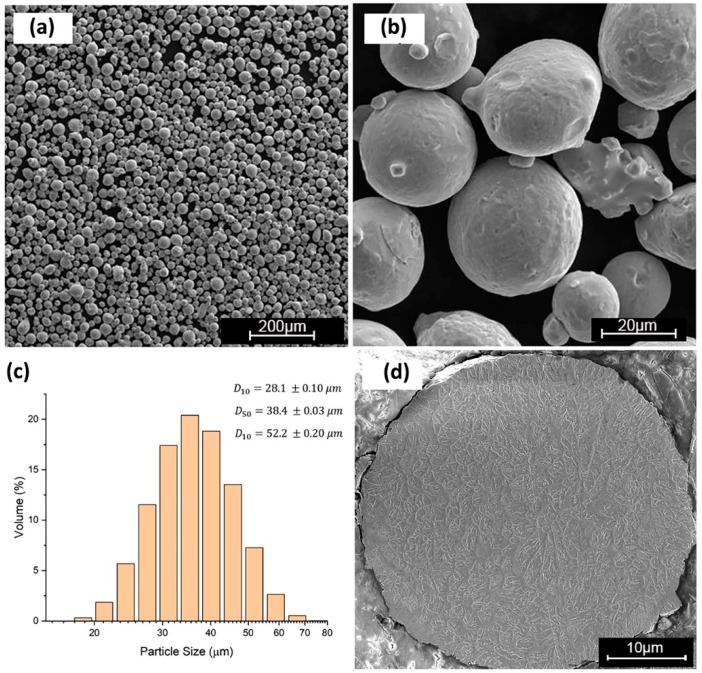
Characteristics of H13 powder: (**a**,**b**) Powder morphology; (**c**) particle size distribution (PSD) analysis; and (**d**) cross-section of the powder particle.

The gas-atomized AISI H13 powder used in this work was supplied by LPW Company (United Kingdom). The particle size distribution (PSD) was measured using the laser diffraction wet method via the Master Sizer 3000 (Malvern, Worcestershire, UK) instrument with the powder dispersed in water. PSD is quantified by D (α), which represents the diameter of the measured particle, where α is the volume percentage of the particles that have a smaller diameter than D. The powder morphology was investigated using a TESCAN VP (TESCAN, Brno, Czech Republic) scanning electron microscope (SEM). Figure 1 shows the morphology and PSD of the powder material used in this work. The chemical composition of the powder used in this study was measured using ICP-OES by digestion. Table 2 shows the chemical composition of AISI H13 powder.

### 2.2. SLM Processing Parameters

**Figure 2:**
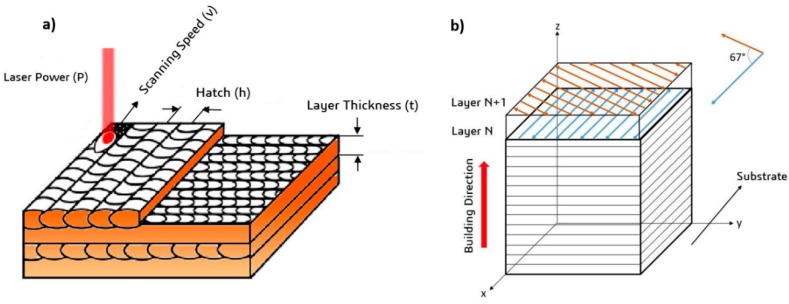
Schematic presentation of selective laser melting (SLM) processing: (**a**) SLM process parameters; and (**b**) scanning strategy.

AISI H13 samples were fabricated using the SLM process on an EOS M280 machine (EOS, Krailling, Germany) equipped with a fiber laser system delivering power levels of up to a maximum of 400 W. An atmosphere of nitrogen gas was applied to reduce the oxygen content in the build chamber to less than 0.1%, hence reducing the oxidation during the melting process. The most important SLM process parameters include laser power, scanning speed, hatch spacing, and layer thickness, as shown in Figure 2a. These parameters can be combined to calculate the volumetric laser energy density using Equation (1).

(1) $$E_{v}=\frac{P}{v\times h\times t}$$

where: *E_v_*: Volumetric laser energy density (J/mm^3^), *P*: Laser power (W), *v*: Scanning speed (mm/s), *h*: Hatch spacing (mm), and *t*: Layer thickness (mm). 

**Figure 3:**
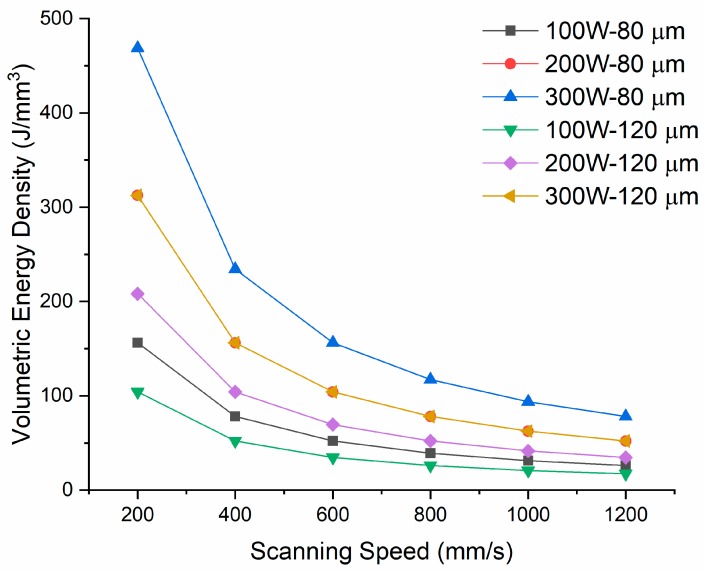
Volumetric energy density variation across the design matrix.

In this study, samples of H13 tool steel with a size of 10 × 10 × 15 mm^3^ were SLM-manufactured using a laser power of 100, 200, and 300 W; scanning speed of 200, 400, 600, 800, 1000, and 1200 mm/s; and hatch spacing of 80 and 120 µm. A full factorial design of experiments (DOE) was adapted to design the experimental matrix. Each run was repeated 3 times. In order to investigate the effect of preheating on the flaw development of the material at hand, the same set of process parameters in the devised DOE was printed applying a preheating of 200 °C to the build plate. The samples were produced directly on the build plate and did not undergo any post-processing procedures. A constant layer thickness of 40 µm, 67° scanning rotation between subsequent layers (Figure 2b), and a stripe scanning strategy were maintained during the process. The contouring, up-skin, and down-skin parameters were deactivated so that only the hatching parameters were considered. Table 3 presents the design of test matrix and Figure 3 shows the calculated volumetric energy density across the design matrix against scanning speed. The devised design matrix encompasses volumetric energy densities ranging from 17.36–465.75 J/mm^3^. The sample codes having the prefix P have been printed with preheating of 200 °C.

### 2.3. Sample Characterization Methods

The density of the as-built samples was measured using Archimedes principle with the aid of a scale with accuracy of ±0.1 mg. The Keyence (Osaka, Japan) VHX series of digital microscopes, a digital optical microscope, was used to investigate the cracks and observable microstructural features. The Alicona G5 “Infinite Focus” (Bruker alicona, Graz, Austria), a focus variation measuring instrument, was used to quantify the surface roughness of the samples. The Verios XHR scanning electron microscope from Thermo-Fisher Scientific (Waltham, MA, USA) was used to perform electron backscatter diffraction (EBSD) and energy-dispersive X-ray spectroscopy (EDS). To detect the phases present in the microstructure of SLM-manufactured samples, X-ray Diffraction (XRD) was carried out using a Bruker D8 DISCOVER (Billerica, MA, USA) with a DAVINCI design diffractometer equipped with a cobalt sealed tube source (wavelength of 1.7902 Å) and a VANTEC-500 area detector. For these measurements, a range of 20–130° 2θ with a step size of 0.01° and an acquisition time of 2 s per increment were used. Pattern analysis was performed with the software DIFFRAC.EVA V3.0 (Billerica, MA, USA).

For microstructural analysis, samples were cut via wire-cut EDM along the build direction. Then, the samples were ground using SiC abrasive papers with a mesh of 600, 800, 1200, 2400, and 4000 followed by a polishing process using a diamond paste of a size 6, 3, and 1 µm. In addition, the samples were chemically etched with 4% nital reagent and evaluated using SEM and a Nikon LV100 optical microscope. For EBSD analysis, the aforementioned procedure of the samples was followed by a 5-min polishing on a chemical resistant cloth with a colloidal silica suspension, and finally a 4-h vibratory polishing with the same suspension. Vickers micro-indentations were made using a load of 200 gf. Six measurements on the polished surface of each sample were carried out and the average was used. Moreover, nano-indentations were made using Anton Paar NHT3 nano-indentation tester (Anton Paar, Graz, Austria), in which the testing parameters were as follows: Maximum load used = 50 mN, loading and unloading rate = 100 mN/min, and the dwell time was 5 s.

## 3. Results and Discussion

**Figure 4:**
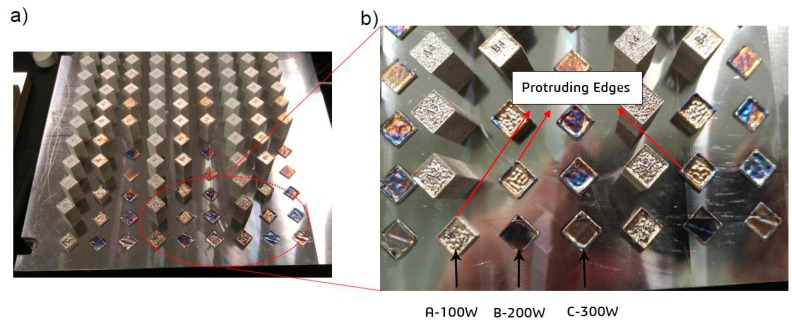
SLM-processed samples: (**a**) Failed parts are within the red ellipse; and (**b**) protruding edges of the failed parts.

The manufacturing process to build the batch of samples took a continuous 22 h to finish; however, as can be observed in Figure 4a, nine samples failed in every three repetitions. The main reason for this failure was the collision of these 10 samples and the powder recoater. The referenced samples were excluded from the batch in the first hour of the printing operation, since this interference could have resulted in imminent damage to the ceramic recoater, a very fragile component of the machine. The root cause of the printing failure of these samples is related to the fact that these samples have high laser energy density, ranging from 150 to 480 J/mm^3^. Thus, on each layer, the molten material accumulates on the border due to heat and mass transport, resulting in a thick solidified “protrusion”, on all four edges of the samples, as indicated in Figure 4b. It is worth mentioning that the same parts, i.e., associated with the volumetric energy densities of above 150 J/mm^3^, also failed under preheating condition.

### 3.1. Density Behavior

**Figure 5:**
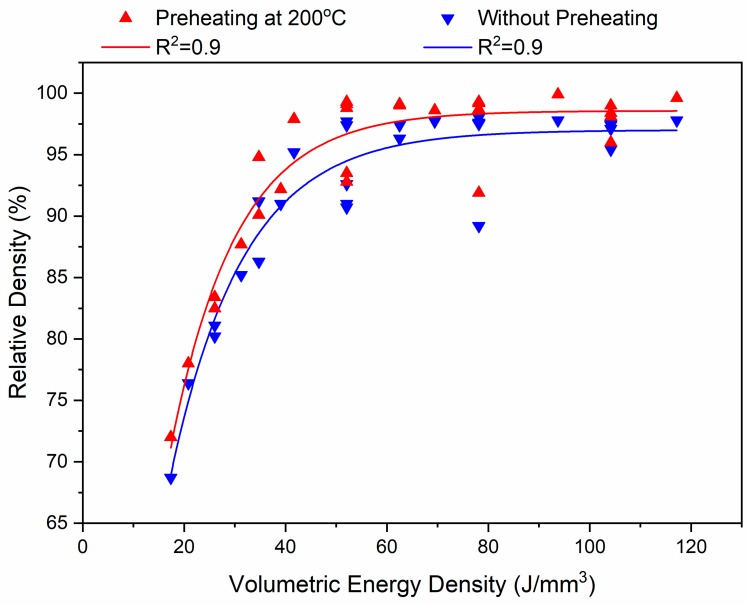
Effect of energy density on the relative density of the parts with and without preheating.

Figure 5 shows the effect of volumetric energy density on the relative density of the SLM-processed H13 parts. With and without substrate preheating of 200 °C, the results showed that increasing the energy density level sharply increases the relative density of the samples in a non-linear fashion up to a value of approximately of 60 J/mm^3^ and then no significant increase can be seen. The increase in relative density was found to be higher for the samples with preheating process than those without preheating. The effectiveness of increasing laser energy density on increasing the relative density has been confirmed for different materials [24,25,26].

Porosity and lack of fusion are common defects that develop during additive manufacturing processes. If not reduced or eliminated, they could adversely affect the mechanical properties of the components [27]. There are a couple of mechanisms enhancing the development of porosity in additive manufacturing. Operating in the keyhole mode associated with higher energy densities, entrapped gas porosities inside the powder particles during the atomization process that leave micro-porosities in the part, interaction of the shielding gas or vaporization products with the melt, and lack of fusion defects that are attributed to low energy inputs insufficient to create full melting [18]. 

**Figure 6:**
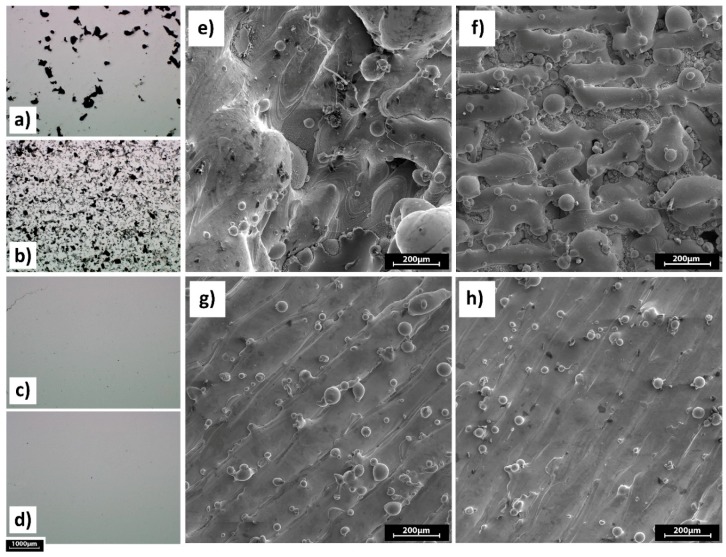
Effect of process parameters on the relative density of the samples, optical micrographs of the cross-sections: (**a**) Sample A2; (**b**) sample A10; (**c**) sample C10; and (**d**) sample C9. SEM micrographs of the top surfaces of samples: (**e**) Sample A2; (**f**) sample A10; (**g**) sample C10; and (**h**) sample C9.

Figure 6 illustrates the influence of individual process parameters in a one-factor-at-a-time (OFAT) manner. Increasing the scanning speed at the same power level has a negative effect on the relative density of the parts because at higher scanning speeds the laser energy is insufficient for complete melting of the powder bed, thereby resulting in a lack of fusion between layers.

This lack of fusion leads to increased pores and voids, which in turn tend to decrease the relative density. As it is shown in Figure 6a,b that corresponds to parts A2 (P = 100 W, v = 200 mm/s, h = 120 µm) and A10 (P = 100 W, v = 1000 mm/s, h = 120 µm) respectively, sample A10 features more porosity due to incomplete melting which is evident in the Figure 6f. Whereas, sample A2 suffers from severe balling shown in Figure 6e. Figure 6c,d correspond to parts C10 (P = 300 W, v = 1000 mm/s, h = 120 µm) and C9 (P = 300 W, v = 1000 mm/s, h = 80 µm), respectively. A comparison between Figure 6a–d clearly shows the influence of increasing laser power on the part quality. In this work, a considerable jump in relative density level was observed in transition from a laser power of 100 W to 200 W. This difference became less significant from 200 W to 300 W. Although hatch spacing is an important factor that could strongly affect the relative density of the SLM-built parts [28], increasing the hatch spacing from 80 and 120 µm brought about no significant difference in the density results. This is because the chosen hatch spacing levels are both equally optimum.

The preheating process can affect the part density, depending on the temperature applied. Upon application of preheating, less heat input is needed from the laser source to melt the powder. That is why, with the use of preheating process, higher scan speeds can be used to produce equally dense parts. In this case, additional post processing may be avoided, thus leading to a more time and cost efficient SLM process [17]. As shown in Figure 5, the differences between the relative density values of the preheated and non-preheated parts are not too high. However, significant differences may be expected on increasing the preheating temperature to higher values.

### 3.2. Cracking Behavior 

**Figure 7:**
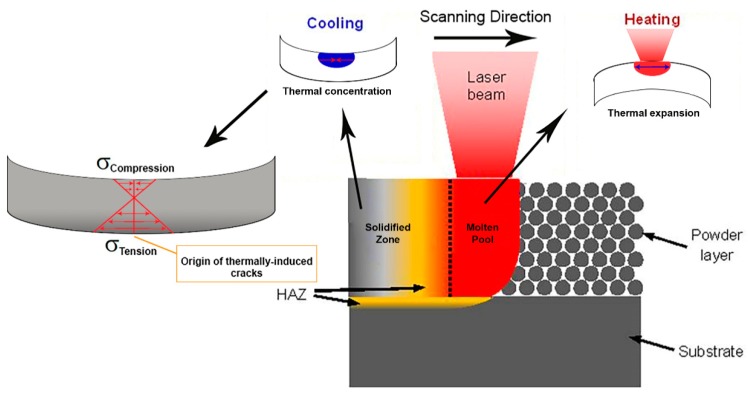
Thermal stresses in SLM and origin of thermally induced cracks (adapted from [29]).

Cracking is another commonly encountered defect in additive manufactured parts that, if not accounted for, could seriously limit parts performance in service. When fabricating H13 tool steel by SLM process, high thermal stresses associated with the process can bring about cracking and delamination from the baseplate. Figure 7 illustrates the mechanism by which thermal stresses can trigger thermally-induced cracking in each layer during the SLM process. 

Due to the high temperature in the upper layers of the solid substrate, these upper layers will expand, while the colder underlying solidified layers will restrict this expansion. This induces compressive stresses in the upper layers of the substrate that may rise above the yield strength of the material and cause plastic upsetting in upper layers. When the yield strength is reached, the compressive stresses cause plastic deformation in the upper layers. When these plastically deformed layers cool down, their compressive state is converted into residual tensile stresses. These residual stresses may induce cracking of the part. Furthermore, the melted top layers tend to shrink due to thermal contraction. This deformation is again prohibited by the underlying layers, thus introducing tensile stresses in the top layers, and compressive stresses below [11]. 

**Figure 8:**
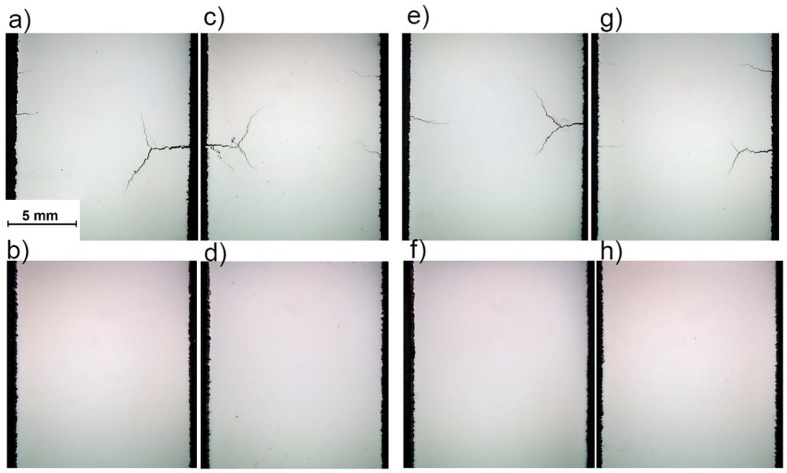
Effect of preheating of 200 °C on dense parts: (**a**) Sample C8; (**b**) sample PC8; (**c**) sample C10; (**d**) sample PC10; (**e**) sample C9; (**f**) sample PC9; (**g**) sample C11; and (**h**) sample PC11.

Figure 8 shows the cracking behavior of some of the parts in the design matrix that already featured acceptable densities. As it is evident from the Figure 8, preheating has proved to be effective in eliminating the cracks.

Preheating the base plate to reduce the steep thermal gradients has been implemented by many researchers. Kempen et al. [11] used a preheating of 200 °C to reduce the extent of cracking and delamination in the case of M2 HSS tool steel. Martens et al. [17] investigated the effect of preheating temperatures of 100, 200, 300, and 400 °C on the SLM-fabricated H13 parts. According to their findings, the residual stresses evolve from compressive at low preheating temperatures to tensile stresses as the preheating temperatures increases. However, better mechanical properties including ultimate tensile strength comparable to those of conventionally fabricated and heat-treated parts were achieved. Krell et al. [19] also investigated the effect of preheating on the properties of SLM-produced H13 tool steel. They found a significant reduction in cracking density by applying preheating of 300 °C. Their results revealed that increasing the preheating temperature will lead into more oxygen uptake in the final parts, which might result in weakening of the mechanical properties.

The total strain developed in the cooling phase has four major contributors, namely elastic (ε_e_), plastic (ε_p_), thermal [ε_T_ = α (T − T_0_)] in which α is the coefficient of thermal expansion; T is the local temperature; and T_0_ is the initial temperature, and phase transformations (ε_PT_) [20]. Application of a preheating temperature reduces the temperature difference at each point resulting the reduction of thermal contribution to the total strain, and ultimately mitigating the residual stresses. The results of the current study revealed that the direction of the density and thermally-induced cracking improvements are opposite. At laser power of 100 W, little, if any, cracks are present but the material suffers from severe porosity. On the other hand, at higher laser powers, the material possesses good density, while suffering from aggravated cracking. Preheating of the build plate proved to be a good way of widening the safe processing windows of H13 tool steel.

### 3.3. Surface Roughness

Surface roughness is one of the most important features of complex geometries produced by AM. There are two main mechanisms bringing about rough surfaces in additively manufactured parts. “Staircase effect” is one of the mechanisms that finds its roots in the stepped approximation by layers of curved and inclined surfaces in complex geometries [30]. In the current study, because the geometry of the build parts are upright coupons, this effect is absent. The other mechanism, which is related to process parameters, is the insufficient melting of the powder particles on the bed and balling phenomenon [31,32]. Surface roughness is measured using a profilometer or analyzing the surface morphology using SEM. On the surface, the height of a peak or the depth of a valley (*f_n_*) is measured at *N* locations along the profile length *L*. Consequently, the average surface roughness (*R_a_*) is calculated using Equation (2) [33] as:

(2) $$R_{a}=\frac{1}{N}\sum_{i=1}^{N}\left|f_{n}\right|$$

**Figure 9:**
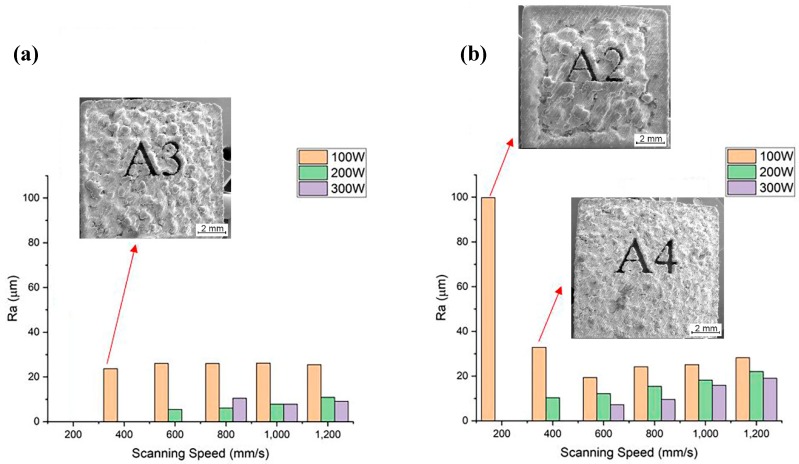
Surface roughness measurements: (**a**) h = 80 µm and (**b**) h = 120 µm.

Figure 9 shows the surface roughness measurements of the samples presented in the design matrix. Samples A3, A2 and A4 (Table 3) suffer from severe balling that leaves big lumps of solidified material on the surface, leading into aggravation of surface roughness. The balling effect and insufficient melting of powders in the case of the SLM-parts processed with a laser power of 100 W at all the scanning speeds used seem to be responsible for featuring rough surfaces. Increasing laser power, particularly from 200 to 300 W, at all hatch spacing and scanning speeds led into mitigation of surface roughness, which is in agreement with the literature [34,35]. 

**Figure 10:**
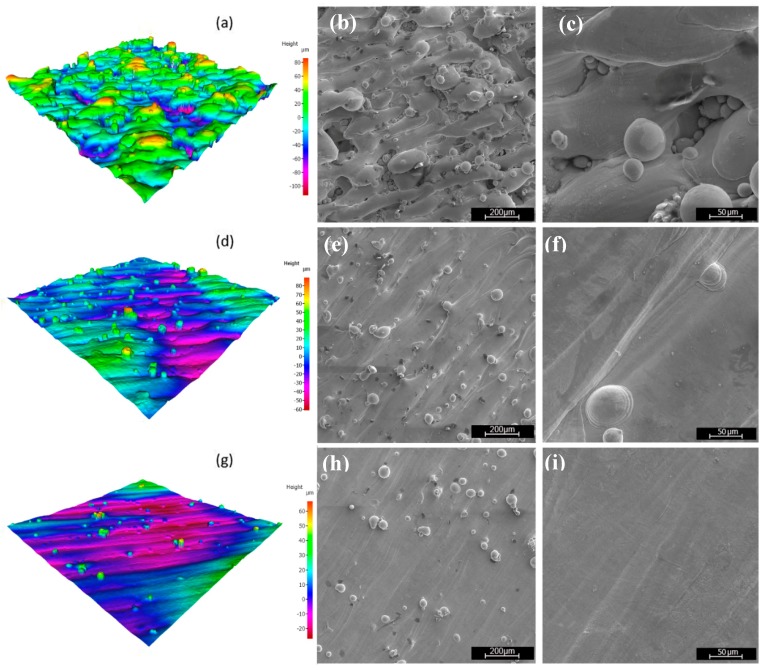
Surface texture scan and SEM micrographs: (**a**–**c**) Sample A9, (**d**–**f**) sample B9, and (**g**–**i**) sample C9.

The decrease in the average roughness may be attributed to the increase in heat input provided by the laser power which in turn yields a wider melt pool causing a better overlap between adjacent scan tracks. Figure 10 shows surface texture scans along with the SEM image and the surface scan of the top surfaces of the samples A9, B9, and C9 built at constant scanning speed of 1000 mm/s and hatch spacing of 80 µm, and a laser power of 100, 200, and 300 W, respectively. The best surface roughness achieved is 6.1 µm. The further improvement of the surface roughness is limited by the large un-melted powders left on the surface (Figure 10e,h). As may be seen from Figure 10, increasing the laser power, when the other process parameters are kept unchanged, generally tends to decrease the surface roughness of SLM-manufactured samples. Preheating of 200 °C used in this study has no substantial effect on quality of the obtained surfaces.

### 3.4. Microstructural Analysis

**Figure 11:**
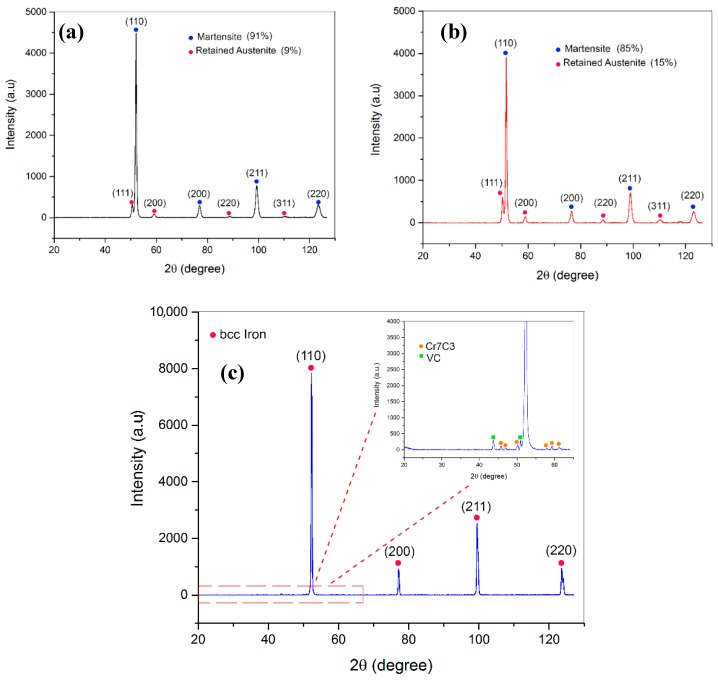
XRD analysis: (**a**) Sample C9 without preheating; (**b**) sample PC9 with 200 °C preheating; and (**c**) as-cast sample.

Figure 11 depicts the XRD analysis of two SLM-manufactured samples as follows: Sample C9 without preheating (Figure 11a) and sample PC9 with preheating of 200 °C (Figure 11b) under the same processing parameters. Both the samples possess a volumetric energy density of 62.5 J/mm^3^. Figure 11c shows the XRD analysis of the as-cast sample, used for a comparison. For the XRD testing, the cross section of the samples along the build direction were polished and then examined. 

It was found that the as-cast sample contains predominantly α-Fe with vanadium and chromium carbides. Generally, all the SLM-fabricated parts across the design matrix revealed the same phases. The microstructures of as-built samples contain martensite (a = 2.8 Å) as the dominant phase and some amount of retained austenite (a = 3.6 Å) for both printing conditions, with and without preheating. This is consistent with the observations from the previously studies [17,22]. Using the Rietveld method, the phase quantification of the XRD patterns was performed. The results showed that the retained austenite content in the matrix of the sample PC9 built with a preheating of 200 °C was about 66% higher than that measured in the matrix of the sample C9 processed without preheating. This may be related with the less fast quenching being occurred with an application of preheating process. Disappearance of the carbides may be attributed to the high cooling rates experienced during the SLM process that significantly restricts the diffusion mechanisms, thereby impeding precipitation of the carbides. However, in conventionally processed H13, these carbides are evident inside and along grain boundaries.

**Figure 12:**
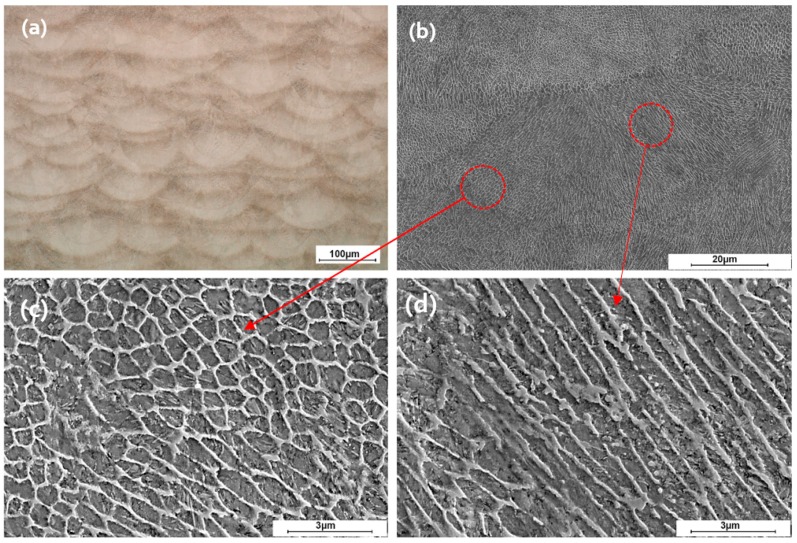
Microstructure of SLM-processed sample in as-built condition along the build direction: (**a**) Optical microscopy of a cross-section; (**b**) SEM micrograph of a melt-pool; (**c**) higher magnification of an area showing fine equiaxed dendrite; and (**d**) higher magnification of an area representing columnar dendrite.

Microstructural analysis was performed on the as-built parts fabricated with the SLM process. Optical microscope observation of etched samples was performed to reveal the melt pool shapes and laser tracks along the build direction (Z direction), as shown in the Figure 12a. There are some oddly shaped melt pools in the micrograph due to scanning direction changes 67° in each layer and therefore the micrograph shows the intersection of the melt pool with the sectioning plane along the build direction. The melt pool cross-sectional view reveals curved morphology which reflects the laser’s Gaussian energy profile. Figure 12b–d illustrate the SEM micrographs of the etched sample through which two types of crystals, produced during the solidification process, may be seen. These are equiaxed (Figure 12c) and columnar crystals (Figure 12c), irregularly distributed within the structure.

XRD and EBSD measurements were carried out to characterize the phases present in the microstructure. Generally, two phases (bright and dark) may be seen. The bright phase was identified to be primarily fcc (austenite) and the darker phase bcc-structured which is martensite. A dominant cell-like morphology is observed for both preheated and non-preheated samples. The size of these bcc-structures cells varies from 600 nm up to 1 µm, whereas the wall thickness of the fcc structures varies from 100 to 200 nm. In some regions in the melt pool, due to different thermal regime, these cell-like microstructures get stretched (Figure 12d) along the direction of maximum thermal gradient.

The results of the microstructural investigation revealed that, other than the amount of martensite and retained austenite, the preheating process of 200 °C has no significant effect on the cellular structure in comparing with that of the non-preheated samples. These results are consistent with those findings on the processing of H13 tool steel cited in [17,19]. Considering high cooling rates experienced in SLM, it is expected to have a fully martensitic microstructure at room temperature. However, a considerable amount of retained austenite is detected using XRD and EBSD methods. Two mechanisms may be adapted to explain the presence of some amount of austenite in the structure of SLM-processed H13. The first is given by Holzweissig et al. [22]. They postulated that SLM process is similar to Quench and Partitioning (Q + P) process in which the martensitic structure evolves as a result of high cooling rates associated with the SLM process. Upon melting of next layer, the previously solidified microstructure gets austenitized and quenched again. In this process, due to diffusion of carbon, which is a strong austenite stabilizer, some austenite is stabilized and remains in the room-temperature microstructure. The second mechanism, suggested by Zhong et al. [36], for the case of SLM-manufactured 316 L stainless steel, is related to a segregation during solidification that accounts for local stabilization of austenite in the microstructure.

**Figure 13:**
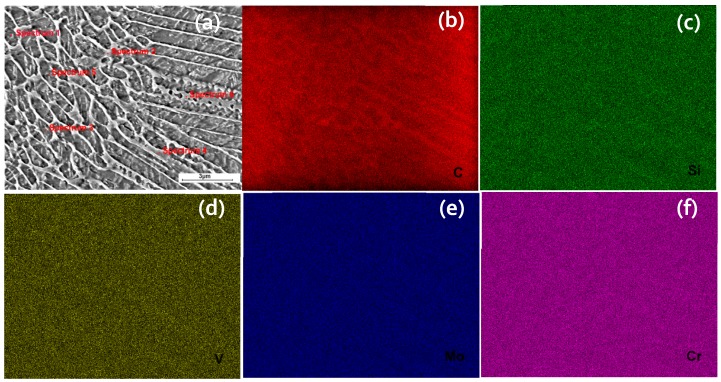
Energy-dispersive X-ray spectroscopy (EDS) maps of the formed morphologies along the build direction: (**a**) Location of points; (**b**) C element; (**c**) Si element; (**d**) V element; (**e**) Mo element; and (**f**) Cr element.

In order to identify the elemental composition of the formed morphologies of SLM-processed H13 tool steel, SEM-EDS analysis was performed on six points, as shown in Figure 13a. Three points were located inside the cells (1, 3, and 5) and the other three were on the walls of the cells (2, 4, and 6). Table 4 presents the composition of the measured points. Figure 13b–f illustrates the EDS maps of the major alloying elements, C, Si, V, Mo, and Cr, respectively. 

As it is evident from the Figure 13b and Table 4, the concentration of C is much higher in the cell walls, where the austenite phase is present, than inside the cell. Such a high carbon concentration may explain the stabilization of austenite phase at room temperature, since C is a strong austenite stabilizer. The other alloying elements (Si, V, Mo, and Cr) are seen from Figure 13c–f and Table 4 to partition about equally inside the cells and in the walls of the cells.

**Figure 14:**
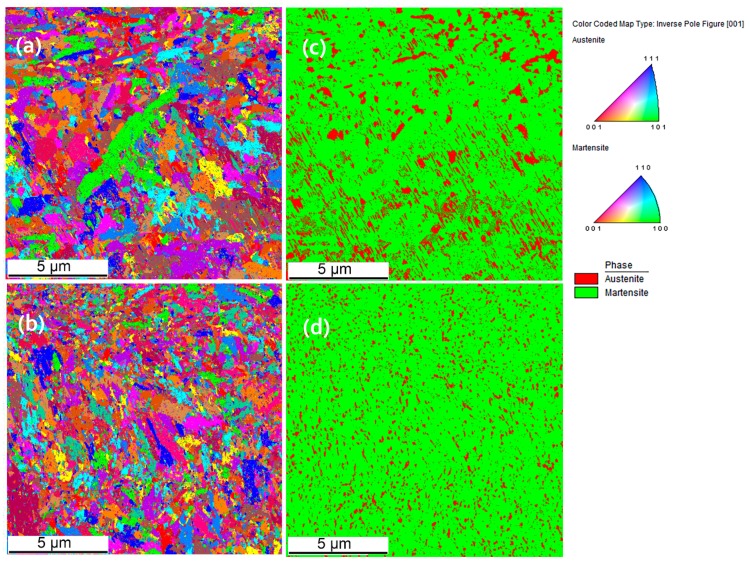
Electron backscatter diffraction (EBSD) grain orientation maps of the sections along the build direction: (**a**) Sample PC9 and (**b**) sample C9. Spatial distribution of phases: (**c**) Sample PC9 and (**d**) sample C9.

Figure 14a,b depict the color maps obtained by electron-backscatter diffraction (EBSD) from cross sections along the build direction (Z) for the samples PC9 and C9 (Table 3). Figure 14c,d show the phases detected by EBSD over a relatively small part of the as-built samples (scan step size: 150 nm). As discussed earlier, a considerable amount of retained austenite is present in the SLM-processed H13 tool steel. Comparing the morphologies of the retained austenite shown in Figure 12 and Figure 14, it is evident that the morphology in the Figure 12 is continuous and that in Figure 14 is found to be discontinuous. The reason behind it is the low amount of certainty index (CI) in the unprocessed data that is related to the high density of dislocations in the microstructure limiting the quality of the Kikuchi patterns. The post-processing of data excludes the data points with low CI, leaving the morphologies discontinuous in the phase maps. The cross sections show a fine grain structure with an average size of 0.52 µm as a result of the rapid solidification. The microstructure when viewed from the side, shows epitaxial columnar grains oriented along the SLM building direction. These grains formed during the solidification of the previous layer elongated toward the building direction of heat conduction through the laser scan with the same orientation. Rotation of the scanning direction by 67° usually breaks up the defined epitaxial columnar structure [37]. 

### 3.5. Hardness 

**Figure 15:**
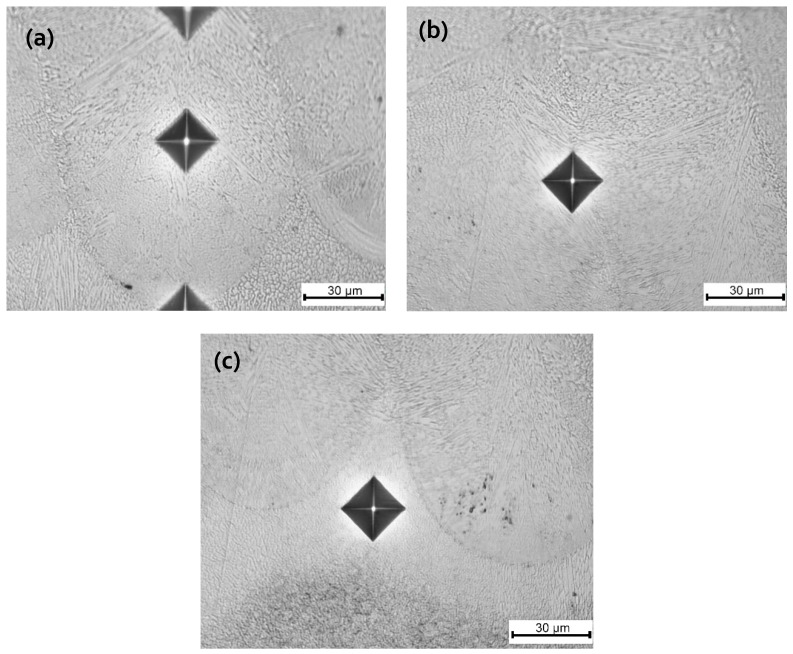
Vickers microhardness indentations in the topmost layer of PC9 sample along the build direction: (**a**) Along the melt-pool; (**b**) re-melted boundary; and (**c**) heat affected zone (HAZ).

Hardness is one of the most commonly used for the characterization of mechanical properties because hardness measurements are quick, relatively inexpensive and provide insight to other properties such as yield strength and wear resistance [38,39]. Micro-hardness tests were conducted on the samples representing different process parameters. No significant difference was found in the hardness of the samples and its value ranged from minimum of 650 to 689 HV 0.2. This may be due to the high cooling rates during the process that leaves the martensitic phase in large portions. Apart from that, the size of the grains also plays a role in the high and uniform values of hardness. Such fine and uniform structures reflect in little differences in the microhardness values of the as-built samples. Figure 15 shows the microhardness indentations made in the top most layer of the PC9 sample, targeting the height of the melt pool (Figure 15a), re-melted region between two adjacent melt-pools (Figure 15b), and heat affected zone (HAZ) region (Figure 15c). On the average, the whole set of points yields 618 HV 0.2 (~56 HRC). The uniform distribution of the hardness in the as-built samples can be attributed to the high amount of martensite phase. 

Local mechanical properties including nano-hardness and Young’s modulus of the as-built samples were measured via nano-indentation. The indenter was a Berkovich diamond three-sided pyramid with a nominal angle of 65.3° and a radius of about 100 nm. The maximum load used was 50 mN. The loading and unloading rate was 100 mN/min and the dwell time was 5 s. The nano-indenter precisely measured the continuous displacement and force of the indenter towards and into the specimen, with displacement and force errors about 1 nm and 1 µN, respectively. The hardness and modulus values were calculated from the load–displacement curve for each indentation using Equation (3) [38]:

(3) $$E_{r}=\frac{S\sqrt{\pi }}{2\beta \sqrt{A}}\;,\;\frac{1}{E_{r}}=\frac{1-v^{2}}{E}+\frac{1-v_{i}^{2}}{E_{i}}$$

where *S*, is the slope of the unloading curve at the maximum depth; *E_r_* is the reduced modulus; *E* and *v* are the Young’s modulus and Poisson ratio of the material, respectively, and *E_i_* and *v_i_* are those for the indenter tip; *β* is a constant dependent on the indenter geometry (*β* = 1.034 for a Berkovich indenter); *A* is the projected contact area, which is a function of contact depth (*A* = 24.5 *h*^2^*_c_*). The relationship between contact depth *h_c_* and the maximum depth, *h_max_* is given in Equation (4) as:

(4) $$h_{c}=h_{max}-h_{d}=h_{max}-\varepsilon \frac{P_{max}}{S}$$

where *h*_d_ is the depth of elastic deflection, *ε* is a constant dependent on the indenter geometry, and *P_max_* is the peak load. The hardness *H* defined as the applied load divided by the projected area of contact can be calculated according to Equation (5) as:

(5) $$H=\frac{P_{max}}{A\left(h_{c}\right)}$$

**Figure 16:**
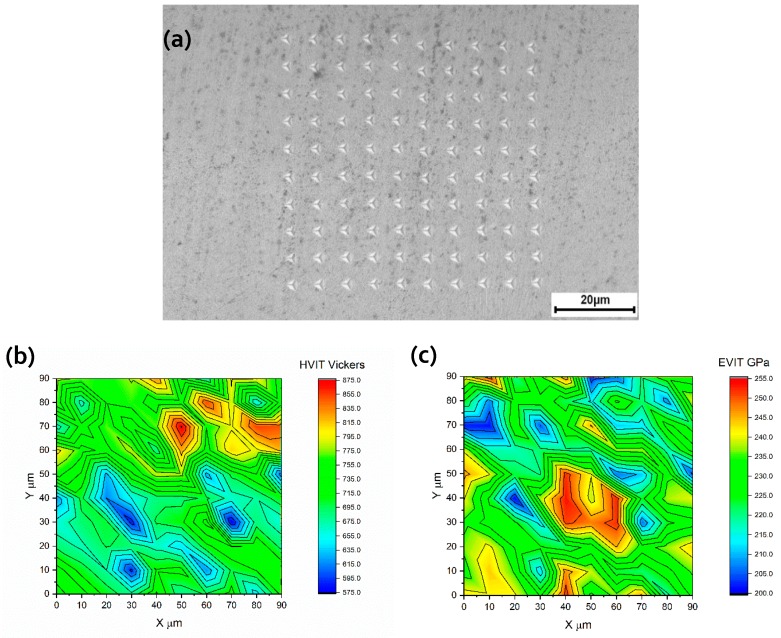
Mechanical properties of the PC9 sample along the build direction: (**a**) Indention array; (**b**) distribution of nano-hardness; and (**c**) Young’s modulus distribution.

Figure 16 shows the distribution of nano-hardness and Young’s modulus in the designated area in the picture located at the surface of the sample PC9 (P = 300 W, v = 1000 mm/s, and h = 80 µm) processed with a preheating temperature of 200 °C. The map contains 100 indentations. As seen in the figure, the greatest nano-hardness values were obtained at the top layers of the sample. This may be attributed to the formation of hard martensite in each newly solidified layer due to high cooling rates during SLM process. The hardness significantly decreased when moving away from the top surface and the lowest hardness was found to be 575. Such a decrease in hardness may be related to the tempering of martensite. These findings are in a good agreement with those found by Mertens et al. [19]. 

## 4. Conclusions 

In this study, samples of H13 tool steel with a size of 10 × 10 × 15 mm^3^ were SLM-manufactured using a laser power of 100, 200, and 300 W; scanning speed of 200, 400, 600, 800, 1000, and 1200 mm/s; and hatch spacing of 80 and 120 µm. A constant layer thickness of 40 µm, 67° scanning rotation between subsequent layers, and a stripe scanning strategy were maintained during the process. A preheating process of 200 °C was considered. The main conclusions can be drawn as follows:

1. The relative density of the as-built material, processed with and without preheating of 200 °C, increased non-linearly with increasing the volumetric energy density up to a value of about 60 J/mm^3^ and then no significant increase was seen.
2. The relative densities of the samples processed with preheating were relatively higher when compared with those of the non-preheated samples; a relative density of 99.7% was achieved.
3. Application of the preheating process, not only enhanced the relative density, but also it helped in eliminating the thermally-induced cracks. In fact, preheating of the base plate broadens the safe processing window of SLM-manufactured H13 tool steel.
4. The best surface roughness achieved in this work was 6.1 µm, corresponding to the sample B5 (P = 200 W, v = 600 mm/s, and h = 80 µm). Due to un-melted powders, further improvements in the surface roughness was not possible and parts need to go through post-processing in case better finishes are required.
5. The microstructure of the as-built samples showed fine equiaxed cellular-dendritic structure (600 nm up to 1µm). All the samples studied showed both a dominant martensite and varying retained austenite contents. The preheating temperature of 200 °C led to an increase in the amount of retained austenite. For example, the amount of retained austenite in a preheating sample PC9 was found to be 66% higher when compared to that of the non-preheated sample C9.
6. The microhardness of the as-built samples was found to vary from 650 to 689 HV 0.2, which is comparable to a conventionally produced H13 tool steel.

## Figures and Tables

**Table 1:** Important research activities around H13 tool steel.

Machine	Process Parameters	Sample Size (mm^3^)	Remarks	Reference
Realizer II ^SLM^ MCP HEK	P = 100 W, v = 200–400 mm/s, point distance = 30 µm, layer thickness = 30 µm, hatch exposure time = 75–100 µs, Preheating = 100–300 °C	5 × 5 × 10	Porosity and density measurements, heat-treatment post processing	[19]
SLM Solutions 250 HL	P = 150 W, v = 300 mm/s, hatch spacing = 50 µm, Preheating of 200 °C,	10 × 10 × 10	Microstructural characterization, residual stress measurements	[20]
SLM Solutions 250 HL	P = 100–300 W, v = 400–1200 mm/s, hatch spacing = 90–150 µm,	10 × 10 × 10	Density optimization with D-optimal design of experiment	[21]
In-house developed SLM	P = 170 W, v = 400, 800 mm/s, hatch spacing = 105 µm, layer thickness = 30 µm	10 × 10 × 10	Influence of preheating at 100, 200, 300, and 400 °C on the status of residual stresses, and mechanical properties	[17]
SLM Solutions 250 HL	P = 175 W, v = 750 mm/s, hatch spacing = 120 µm, layer thickness = 30 µm.	8 × 3 × 1.5	Microstructural characterization and interrelationship between process parameters and microstructural evolution	[22]
SLM Solutions 250 HL	P = 125–375 W, v = 289–2604 mm/s, hatch spacing = 120 µm, Layer thickness = 30 µm	4 × 4 × 4	Density optimization and manufacturability of self-supporting conformal cooling channels	[5]
EOS M280 SLM	P = 280 W, v = 980 mm/s, hatch spacing = 120 µm, layer thickness = 40 µm.	10 × 10 × 3	Microstructural characterization and effect of post-processing heat treatment on the microstructure of the as-build samples	[23]

**Table 2:** Chemical composition of AISI H13 powder.

Element (wt %)	Cr	Mo	Si	V	Mn	C	Fe
ASTM-A681	4.75–5.5	1.10–1.75	0.8–1.25	0.8–1.2	0.2–0.6	0.32–0.45	Bal.
Reported by Supplier	5.36	1.38	1.12	1.05	0.42	0.39	Bal.
ICP-OES	5.27	1.34	1.08	0.97	0.40	0.39	Bal.

**Table 3:** Design of experiments implemented in this work.

Sample Code	P (W)	v (mm/s)	h (µm)	Sample Code	P (W)	v (mm/s)	h (µm)	Sample Code	P (W)	v (mm/s)	h (µm)
A1	100	200	80	B1	200	200	80	C1	300	200	80
A2	100	200	120	B2	200	200	120	C2	300	200	120
A3	100	400	80	B3	200	400	80	C3	300	400	80
A4	100	400	120	B4	200	400	120	C4	300	400	120
A5	100	600	80	B5	200	600	80	C5	300	600	80
A6	100	600	120	B6	200	600	120	C6	300	600	120
A7	100	800	80	B7	200	800	80	C7	300	800	80
A8	100	800	120	B8	200	800	120	C8	300	800	120
A9	100	1000	80	B9	200	1000	80	C9	300	1000	80
A10	100	1000	120	B10	200	1000	120	C10	300	1000	120
A11	100	1200	80	B11	200	1200	80	C11	300	1200	80
A12	100	1200	120	B12	200	1200	120	C12	300	1200	120

**Table 4:** EDS analysis, composition (wt %) of the tested points.

Element	Point 1	Point 2	Point 3	Point 4	Point 5	Point 6
C	6.17	7.42	5.84	7.58	6.17	7.24
Si	0.92	1.01	1.01	0.9	0.99	1.02
V	1.06	1.00	1.13	1.01	0.95	1.14
Mo	1.29	1.53	1.61	1.62	1.69	1.67
Cr	5.15	5.28	5.12	5.4	5.09	5.34
Fe	85.41	83.76	85.29	83.49	85.11	83.59
Total	100	100	100	100	100	100
